# Assessment of Knowledge, Awareness, and Attitude Regarding Human Papillomavirus Vaccine Among Young Tribal Women in India

**DOI:** 10.7759/cureus.85746

**Published:** 2025-06-11

**Authors:** Yukta Sain, Havilah Twinkle Reddipogu, Chandini Kollabathula, Mohammed Jaffer Pinjar

**Affiliations:** 1 Physiology, Great Eastern Medical School and Hospital, Srikakulam, IND; 2 Physiology, Maharaja’s Institute of Medical Sciences, Vizianagaram, IND; 3 Physiology, Government Medical College, Rajamahendravaram, IND; 4 Physiology, All India Institute of Medical Sciences, Deoghar, IND

**Keywords:** cervical cancer, hpv vaccine, rural health, tribal women, vaccine awareness

## Abstract

Introduction: Cervical cancer remains a leading cause of morbidity and mortality among women in India, with human papillomavirus (HPV) identified as the principal causative agent. Despite the availability of effective vaccines, uptake remains low, particularly in rural and tribal populations. This study aimed to assess the knowledge, awareness, and attitude regarding HPV vaccination among young tribal women in Andhra Pradesh, India.

Methods: This cross-sectional observational study was conducted among 350 tribal women aged 18-25 years in Ragolu, Srikakulam, during October-November 2023. Participants were selected using simple random sampling. Data were collected via a prevalidated questionnaire in the local language and English, assessing sociodemographic details, knowledge of cervical cancer and HPV, awareness of the HPV vaccine, and attitudes regarding vaccination. Descriptive statistics were used for data analysis.

Results: Only 26% of participants were aware of cervical cancer, and 20.5% had heard of HPV. Among those aware of HPV, fewer than half knew it was sexually transmitted or that vaccines are available for prevention. Awareness of the recommended age and dosing regimen for the HPV vaccine was notably low. Attitudes toward vaccination were generally positive: 69.4% believed HPV vaccination is necessary, and 70.3% were willing to receive the vaccine. However, cost was a barrier, with 52% unwilling to pay 5000-10,000 rupees for vaccination. A majority (90%) expressed a desire for more information about the vaccine, and 87.1% would recommend it to others. Most participants (58.9%) favored including HPV vaccination in the regular immunization schedule.

Conclusion: There is a significant gap in knowledge and awareness of HPV, cervical cancer, and HPV vaccination among young tribal women. Despite this, attitudes toward vaccination are favorable, suggesting that targeted educational campaigns and integration of the HPV vaccine into national immunization programs could substantially improve vaccine uptake and reduce the burden of cervical cancer in underserved populations.

## Introduction

Cervical cancer is a significant public health concern in India, with high incidence and mortality rates among women [1]. According to the HPV Information Centre, India accounts for nearly one-fourth of the world's cervical cancer cases, with approximately 123,907 new cases and 77,348 deaths reported annually [2]. The disease is primarily caused by persistent infection with high-risk types of human papillomavirus (HPV), particularly types 16 and 18, which are responsible for about 70% of all cervical cancer cases worldwide [3]. HPV vaccination represents a critical preventive strategy against cervical cancer. The World Health Organization recommends HPV vaccination for girls aged nine to 14 years before sexual debut [4]. Despite the availability of effective vaccines, their uptake in India remains suboptimal, especially in rural and tribal communities where the burden of cervical cancer is often highest [5].

Several barriers impede widespread HPV vaccination in India, including limited awareness, misconceptions about vaccine safety and efficacy, cultural taboos surrounding sexually transmitted infections, and financial constraints [6]. Understanding the current levels of knowledge, awareness, and attitudes regarding the HPV vaccine among vulnerable populations is essential for designing targeted interventions to enhance vaccine acceptance and coverage. Tribal communities in India, characterized by geographical isolation, limited access to healthcare, and distinct sociocultural practices, face unique challenges in adopting preventive health measures [7]. The health literacy levels in these communities are often lower than the national average, potentially contributing to reduced awareness about HPV and cervical cancer prevention [8].

Previous studies have examined HPV vaccine awareness in urban populations and among healthcare professionals in India [9,10]. However, research focusing specifically on tribal women remains scarce. This study aims to fill this gap by assessing the knowledge, awareness, and attitudes regarding HPV vaccination among young tribal women in Andhra Pradesh, one of the states with a significant tribal population in India. The findings of this study will contribute to the development of culturally sensitive educational interventions and policy recommendations to improve HPV vaccine uptake in tribal communities, ultimately reducing the disproportionate burden of cervical cancer in these underserved populations.

## Materials and methods

Study design and setting

This cross-sectional observational study was conducted in Ragolu, Srikakulam district of Andhra Pradesh, India. The region is predominantly inhabited by tribal communities with limited access to healthcare facilities and health information. The study was conducted between October and November 2023.

Study population

The study included tribal women aged 18 to 25 years who were able to communicate in the local language or English and willing to participate. This age group was selected as they represent potential recipients or recent recipients of the HPV vaccine and are at a stage where preventive health behaviors are being established. Women with previous diagnoses of cervical cancer or those who had already received the HPV vaccine were excluded from the study to avoid bias in knowledge assessment.

Sample size and sampling

A total of 350 participants were selected using simple random sampling from a population list obtained from local health centers and community leaders. The sample size was calculated using the following formula:

$n = \frac{Z&sup2;p(1-p)}{d&sup2;}$

where *Z* is the standard normal variate (1.96 at 5% type I error), *p* is the expected proportion in the population based on previous studies (assumed as 50% due to lack of prior data in this specific population), and *d* is the absolute error (5%). The calculated sample size was 384, which was adjusted to 350 due to logistical constraints and expected nonresponse.

Data collection tool

Data were gathered using a prevalidated questionnaire available in both the local language (Telugu) and English (Appendices). The questionnaire was developed based on a comprehensive literature review [5,8] and was validated through expert review and pilot testing among 30 women with similar characteristics to the study population. The questionnaire assessed four main domains: sociodemographic characteristics, knowledge of cervical cancer and HPV, awareness of the HPV vaccine, and attitudes toward HPV vaccination.

Data collection procedure

Trained female research assistants fluent in the local language administered the questionnaire through face-to-face interviews. For illiterate participants, questions were read aloud, and responses were recorded by the interviewers. The questionnaire consisted of 25 yes/no questions, with 14 focused on knowledge and awareness and 11 on attitudes toward HPV vaccination. Each interview lasted approximately 20 to 30 minutes and was conducted in a private setting to ensure confidentiality.

Data analysis

Descriptive statistics, including frequencies, percentages, means, and standard deviations, were calculated using IBM SPSS Statistics for Windows, Version 25 (Released 2017; IBM Corp., Armonk, New York).

Ethical considerations

Institutional ethical committee approval was obtained prior to study initiation. Informed consent was secured from all participants after explaining the study's purpose, procedures, potential risks, and benefits. Participation was voluntary, and confidentiality was maintained throughout the research process. No personal identifiers were collected, and data were stored securely with access restricted to the research team.

## Results

Sociodemographic characteristics

The mean age of participants was 21.3 ± 2.4 years. Most participants (68.3%) had completed secondary education, while 18.6% had primary education only, and 13.1% had no formal education. The majority (78.9%) were unmarried, and 62.3% reported a monthly family income below 5000 rupees.

Knowledge and awareness

Only 26% of participants were aware of cervical cancer, and a mere 20.5% had heard of HPV. Among those aware of HPV (n = 72), less than half (46.8%) could identify it as sexually transmitted, and only 38.9% knew that vaccines are available for prevention. Knowledge of the recommended age for vaccination and dosing regimens was particularly low, with only 15.3% of those aware of the vaccine knowing the correct age for vaccination, and 11.1% aware of the required number of doses (Table 1).

**Table 1:** Knowledge and awareness of HPV and cervical cancer among tribal women * Percentages calculated from those aware of HPV, n = 72. HPV: human papilloma virus.

Knowledge Item	Frequency (n=350)	Percentage (%)
Aware of cervical cancer	91	26.0
Aware of HPV	72	20.5
Know HPV is sexually transmitted*	34	46.8
Aware of HPV vaccine*	28	38.9
Know correct age for vaccination*	11	15.3
Know correct number of doses*	8	11.1

Attitudes toward vaccination

**Figure 1:**
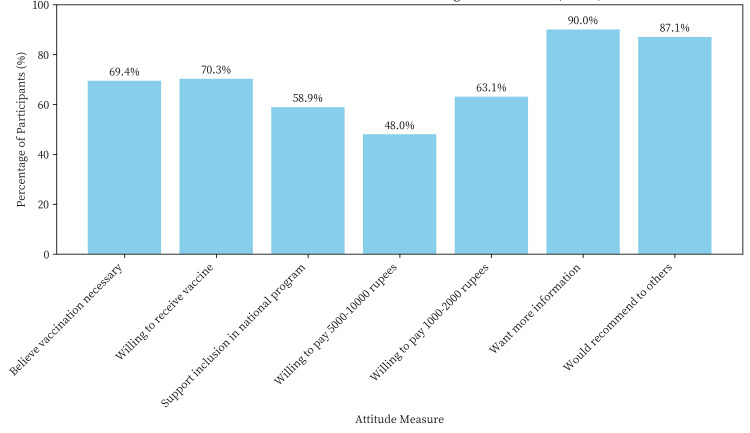
Attitudes toward HPV vaccination among tribal women (n = 350)

Despite limited knowledge, attitudes toward HPV vaccination were generally positive. Most participants (69.4%) believed that HPV vaccination is necessary, and 70.3% expressed willingness to receive the vaccine if offered. A majority (58.9%) supported the integration of the HPV vaccine into the national immunization schedule. However, cost emerged as a significant barrier, with 52% of participants unwilling to pay between 5000 and 10,000 rupees for the vaccination course. When asked about a more affordable price range (1000 to 2000 rupees), the willingness to pay increased to 63.1%. An overwhelming majority (90%) of participants expressed a desire for more information about the HPV vaccine, and 87.1% indicated they would recommend the vaccine to others if provided with adequate information (Figure 1). When asked about preferred sources of health information, healthcare providers (76.3%), community health workers (68.6%), and television/radio (45.7%) were the most frequently cited.

## Discussion

This study reveals a significant gap in knowledge and awareness regarding HPV, cervical cancer, and HPV vaccination among young tribal women in Andhra Pradesh, India. Only about one-fourth of participants were aware of cervical cancer, and even fewer had heard of HPV or the available vaccines. These findings align with previous research conducted in other parts of India and among different population groups [11-14]. A study by Rashid et al. [5] among college students in Delhi reported higher awareness levels (about 66% for cervical cancer and 15% for HPV) compared to our findings, highlighting the disparity in health information access between urban and tribal populations. Similarly, research by Gollu and Gore [9] among medical students showed substantially higher awareness (89% for HPV), underscoring the knowledge gap between healthcare professionals and the general population, particularly in tribal areas.

The low awareness of HPV as a sexually transmitted infection and its causal relationship with cervical cancer indicates a critical need for basic health education in these communities. This lack of awareness may be attributed to several factors, including limited access to healthcare services, low literacy rates, and cultural taboos surrounding discussions of sexually transmitted infections [15,16]. Despite the limited knowledge, the positive attitude toward vaccination observed in this study is encouraging. Most participants believed in the necessity of vaccination and expressed willingness to receive the vaccine if offered. This finding is consistent with research by Khanna et al. [7] in Odisha, where women demonstrated positive attitudes toward cervical cancer screening despite low awareness levels.

Cost emerged as a significant barrier to vaccine acceptance, with more than half of the participants unwilling to pay the current market price for the HPV vaccine. This financial constraint has been identified in previous studies as a major obstacle to HPV vaccine uptake in low- and middle-income countries [17]. The introduction of India's first domestically produced HPV vaccine, "Cervavac," at a potentially lower price point offers hope for improved accessibility [18]. The strong desire for more information about the HPV vaccine expressed by participants suggests that educational interventions could significantly enhance vaccine acceptance and uptake. Healthcare providers and community health workers were identified as preferred sources of health information, highlighting their potential role in disseminating accurate information about HPV and cervical cancer prevention.

The findings of this study have important implications for public health policy and practice. First, there is a need for culturally sensitive and linguistically appropriate educational campaigns targeting tribal communities to improve awareness about HPV, cervical cancer, and the benefits of vaccination. Second, the integration of the HPV vaccine into the national immunization program, as supported by most participants, could address financial barriers and normalize the vaccine as part of routine preventive care. Third, training community health workers to serve as vaccine advocates could leverage existing trust relationships within tribal communities.

Several limitations should be considered when interpreting the results of this study. The cross-sectional design precludes causal inferences and assessment of changes in knowledge or attitudes over time. The self-reported nature of the data may be subject to social desirability bias. Additionally, the study was conducted in one tribal area, potentially limiting generalizability to other tribal populations with different sociocultural characteristics.

## Conclusions

This study reveals a significant knowledge gap regarding HPV, cervical cancer, and HPV vaccination among young tribal women in Andhra Pradesh, India. Despite limited awareness, attitudes toward vaccination are generally positive, suggesting that educational interventions could substantially improve vaccine acceptance and uptake in this population. Addressing financial barriers through policy measures such as vaccine subsidies or integration into the national immunization program, combined with targeted health education campaigns delivered through trusted sources like healthcare providers and community health workers, could significantly enhance HPV vaccine coverage in tribal communities. Such efforts are essential to reduce the disproportionate burden of cervical cancer among women in underserved populations and contribute to India's commitment to eliminating cervical cancer as a public health problem.

## Appendices

**Table 2:** HPV vaccine awareness questionnaire

Sl.No	Questions	Yes
1	Are you aware of cervical cancer?	91
2	Do you know the cause for Cervical cancer?	49
3	Do you know the mode of transmission of cervical cancer?	44
4	Can cervical cancer be identified early?	32
5	Are there any preventive methods against cervical cancer?	46
6	Have you heard of Human Papilloma virus (HPV)?	72
7	Is HPV infection sexually transmitted?	34
8	Do you know that 50% of sexually active women can be infected with HPV?	39
9	Have you heard of any Vaccines for preventing cancer?	36
10	Have you heard of anti HPV vaccine?	28
11	Do you know that the right age for vaccine is between 9 and 26 years?	11
12	Do you know that recommended dose for vaccine is 2 or 3 doses?	08
13	Do you know that other cancers (oral, pharyngeal) caused by HPV can be prevented by HPV vaccine?	15
14	Do you think males also should be vaccinated?	151
15	Do you think it is necessary for you to take the vaccine?	243
16	Do you feel only sexually active ladies should get the vaccine?	95
17	Do you think getting vaccinated depends on the cost of the vaccine?	212
18	Will you be willing to take the vaccine to prevent HPV cervical cancer?	246
19	Will you feel embarrassed to ask for the vaccine?	285
20	Even if there are few risks will you be willing to take the vaccine?	166
21	Will you be willing to take the vaccine if the cost per vaccine is between 5000 -10000 rupees?	168
22	Will you be willing to take the vaccine if the cost per vaccine is between 1000 -2000 rupees?	221
23	Are you willing to get more information about the vaccine?	315
24	Are you willing to recommend vaccine to others if you find it useful?	305
25	Do you think HPV vaccine should be a part of regular vaccination schedule?	206
